# Diagnostic approach for myocardial contusion: a retrospective evaluation of patient data and review of the literature

**DOI:** 10.1007/s00068-020-01305-4

**Published:** 2020-01-25

**Authors:** Esther M. M. Van Lieshout, Michael H. J. Verhofstad, Dirk Jan T. Van Silfhout, Eric A. Dubois

**Affiliations:** 1 Trauma Research Unit Department of Surgery, Erasmus MC, University Medical Center Rotterdam, P.O. Box 2040, 3000 CA Rotterdam, The Netherlands; 2 Department of Cardiology, Erasmus MC, University Medical Center Rotterdam, P.O. Box 2040, 3000 CA Rotterdam, The Netherlands

**Keywords:** Echocardiography, Electrocardiography, Myocardial contusion, Specificity, Troponin

## Abstract

**Purpose:**

Myocardial contusion can be a life-threatening condition in patients who sustained blunt thoracic trauma. The diagnostic approach remains a subject of debate. The aim of this study was to determine the sensitivity and specificity of echocardiography, electrocardiography, troponins T and I (TnT and TnI), and creatine kinase muscle/brain (CK-MB) for identifying patients with a myocardial contusion following blunt thoracic trauma.

**Methods:**

Sensitivity and specificity were first determined in a 10-year retrospective cohort study and second by a systematic literature review with meta-analysis.

**Results:**

Of the 117 patients in the retrospective study, 44 (38%) were considered positive for myocardial contusion. Chest X-ray, chest CT scan, electrocardiograph, and echocardiography had poor sensitivity (< 15%) but good specificity (≥ 90%). Sensitivity to cardiac biomarkers measured at presentation ranged from 59% for TnT to 77% for hs-TnT, specificity ranged from 63% for CK-MB to 100% for TnT. The systematic literature review yielded 28 studies, with 14.5% out of 7242 patients reported as positive for myocardial contusion. The pooled sensitivity of electrocardiography, troponin I, and CK-MB was between 62 and 71%, versus only 45% for echocardiography and 38% for troponin T. The pooled specificity ranged from 63% for CK-MB to 85% for troponin T and 88% for echocardiography.

**Conclusion:**

The best diagnostic approach for myocardial contusion is a combination of electrocardiography and measurement of cardiac biomarkers. If abnormalities are found, telemonitoring is necessary for the early detection of life-threatening arrhythmias. Chest X-ray and CT scan may show other thoracic injuries but provide no information on myocardial contusion.

**Electronic supplementary material:**

The online version of this article (10.1007/s00068-020-01305-4) contains supplementary material, which is available to authorized users.

## Background

Myocardial contusion describes a condition of bruising or (microscopically small) hemorrhaging of the heart muscle caused by blunt thoracic trauma. In patients who have sustained blunt thoracic trauma, the prevalence of myocardial contusion ranges from 0 to 76%, depending on the diagnostic criteria used [1–10].

The bruising is generally caused by a decelerating force on the anterior side of the thorax [11–14]. First, the heart is abruptly pressed to the dorsal side of the sternum causing a bruise on the anterior side (‘coup’). Depending on the amount of energy that needs and can be absorbed by the rib cage, the thoracic spine can hit the heart at the posterior side, resulting in a second bruise (‘contrecoup’). In a final stage, the distance between the sternum and spine will reduce further, resulting in septal or intracardiac structural injuries.

The absence of a clear definition and the fact that there is no accepted gold standard in complementary tests makes diagnosing myocardial contusion difficult. The diagnostic approach of myocardial contusion as well as its clinical course remain subject to debate, because of heterogeneity in clinical presentation and the unpredictable natural course [1, 15–20]. The decelerating force can not only lead to mechanical cardiac injuries, such as rupture of atria or chordae, the bruising may also lead to other cardiac adverse events, varying from mild arrhythmias like premature ventricular complexes, to atrial fibrillation or ventricular fibrillation [2–5, 11–16]. The vast majority of patients who develop arrhythmia after a myocardial contusion do so within 24 h after trauma [13, 21–25]. Whereas on admission to the emergency department a large proportion of patients who sustained blunt thoracic trauma do not show cardiac symptoms indicative of myocardial contusions, clinicians should be prepared for rapid changes in clinical condition of such patients as severe arrhythmia or even cardiac arrest can occur within 72 h [26, 27].

Myocardial contusion is often accompanied by significant extracardiac injuries which may have hemodynamic effects, thereby hampering the possible diagnosis of a myocardial contusion. Patients with hemodynamic changes but without a clear bleeding or cardiac tamponade are very suspect for myocardial contusion [8, 14, 24]. On the other hand, pulmonary contusion, a sternum fracture, or multiple rib fractures should warn the treating physician to be aware of a possible myocardial contusion. Therefore, the relevance of complete diagnostics should not be underestimated after typical trauma mechanisms, even when the first impression on patient’s clinical condition does not indicate severe injury.

Although several diagnostic tests are available, none of them have shown sufficient diagnostic accuracy for diagnosing myocardial contusion [19, 22, 28–32]. Especially for patients who sustained a high-energy trauma but have no clear symptoms or signs (yet), selecting those who require careful observation or telemonitoring from those who can go home safely, is paramount. Echocardiography and electrocardiography may indicate damage to the tissue architecture and subsequent complications, but information on specific cellular damage within the heart muscle requires measurement of cardiac muscle-specific proteins such as troponin T (TnT), troponin I (TnI), or creatine kinase muscle and brain isoenzyme (CK-MB) [2, 28, 29, 33–39]. Since tissue damage cannot occur without cellular damage, whereas the opposite can, a combination of tests is commonly performed.

However, no consensus exists in the optimal diagnostic workup for patients with a possible myocardial contusion. To develop a diagnostic protocol, more insight into the diagnostic properties of the tests available is needed. Therefore, the aim of this study was to determine sensitivity and specificity of echocardiography, electrocardiography, troponins T and I, and CK-MB to identify patients with a myocardial contusion following blunt thoracic trauma. This was done both in a retrospective cohort and by systematic literature review with a meta-analysis.

## Methods

### Retrospective cohort study

Patients presented to a level I trauma center with a suspected myocardial contusion after blunt force thoracic trauma between January 1, 2007, and June 30, 2017, were considered eligible for this single-center retrospective cohort study. The study was exempted by the local Medical Research Ethics Committee.

Potentially eligible patients (i.e., patients for whom myocardial contusion could have been considered) were identified using two strategies. Searching electronic hospital discharge letters and correspondence to General Practitioners that mentioned myocardial contusion (or any synonym possible) resulted in a list of admitted and non-admitted patients. Patients who were admitted to the hospital were also identified from the National Trauma Registry by searching for patients with a registered Abbreviated Injury Score (AIS) for any myocardial injury. The AIS-1998 codes were 441099.1, 441002.1, 441004.1, 441006.4, 441008.3, 441010.3, 441012.5, 441014.6, 441016.6, 441018.6, 441200.5, and 441300.5. The AIS-2005 codes were 441089.9, 441099.1, 441002.1, 441004.1, 441006.4, 114008.3, 441010.3, 441012.5, 441013.5, 441014.6, 441016.6, 441018.6, 441200.5, 441300.5, and 440400.5. Two authors (DJTVS and EAD) identified the eligible patients from these lists. All patients in whom a myocardial contusion was considered were included. Consideration of a myocardial contusion had to be clear from the physician’s notes in the patient’s medical files and could due to results of anamnesis or diagnostic tests. Exclusion criteria were (1) no suspicion of myocardial contusion; (2) trauma mechanism other than blunt trauma; (3) confirmed (non)-ST elevation myocardial infarction [(non-)STEMI]; (4) no diagnostic or outcome data available.

Patient characteristics, injury characteristics, results from patient history, physical examination, diagnostic tests (chest CT-scan, chest X-ray, electrocardiography, echocardiography, and levels of cardiac biomarkers) were collected from medical records. During hospital stay, relevant findings during clinical and telemetric observation, electrocardiography, transthoracic echocardiography, levels of cardiac biomarkers, cardiac adverse events, mortality, and surgical interventions were also collected from the patient’s medical files. Patient characteristics included gender, age at trauma, comorbidities, use of medication that either mask or cause cardiac arrhythmia, and renal function (i.e., eGFR at admission). Thoracic injury characteristics are rib fractures, hemothorax, pneumothorax, cardiac valve defects, sternum fracture, flail chest, pulmonary contusion, and aorta dissection. Details on patient history and physical examination were complaints of chest pain, palpitations, dyspnea, fainting, cardiac murmurs, cardiac rubbing, oxygen saturation, heart rate, systolic and diastolic blood pressure, and Glasgow Coma Score.

Diagnostic tests were checked for signs of abnormalities. For chest CT and X-ray, suspicion of the presence of pericardial effusion was collected from the radiology report. Electrocardiograms (ECGs) were reviewed by a cardiologist (EAD) for signs of ST elevation, ST depression, T-wave inversion, arrhythmia, or intraventricular conduction abnormalities. Transthoracic echocardiography was reviewed (by EAD) for signs of regional wall motion abnormalities, pericardial effusion, or mechanical abnormalities. No transesophageal echocardiography was performed.

Thresholds for increased levels of cardiac biomarkers were 30 ng/L for normal-sensitive troponin T (used until December 31, 2012) and 14 ng/L for high-sensitive troponin T (used since January 1, 2013). Cut-off values for creatine kinase MB (CK-MB) were 7.6 µg/L for men and 4.7 µg/L for women.

Relevant findings during clinical observation with or without telemetry, levels of cardiac biomarkers, electrocardiography, echocardiography, and cardiac adverse events that might suggest myocardial contusion were registered. Relevant cardiac adverse events were arrhythmias (i.e., atrial fibrillation or flutter, premature ventricular complexes, and supraventricular tachycardia), hypotension, and cardiogenic shock requiring inotropic support. In addition, data on relevant surgical interventions, its outcome, and mortality were collected.

Based upon all available data and final judgement of the treating physician as mentioned in the patient’s medical files, patients were categorized as having had a myocardial contusion or not. Diagnostic tests were also categorized as positive or negative for myocardial contusion. No single test could serve as a gold standard for the diagnosis. Signs indicative of myocardial contusion are (1) elevated cardiac biomarkers; (2) new valve defects, regional wall motion abnormalities, pericardial effusion, or other anatomical defects seen on echocardiography; (3) intraventricular conduction abnormalities, atrial fibrillation, premature ventricular complexes, and supraventricular tachycardia seen on electrocardiography.

Data were analyzed using the Statistical Package for the Social Sciences version 24.0 (SPSS, Chicago, IL, USA) and MedCalc (https://www.medcalc.org/calc/diagnostic_test.php). Patients with versus patients without myocardial contusion were compared. Normality of continuous data was tested with the Shapiro–Wilk test, which showed that all were non-normally distributed. Continuous data are shown as median with quartiles and categorical data are shown as numbers with percentage. Statistical significance between the two groups was assessed using a Mann–Whitney *U* test for continuous data and a Chi-square test or Fisher exact test for categorical data. A two-sided *p* value < 0.05 was used as threshold of statistical significance. For each diagnostic test, myocardial contusion prevalence, sensitivity, and specificity were calculated and are reported as percentage with 95% confidence.

### Literature review

A literature search was performed on November 11, 2018, using Embase.com, Medline Ovid, Web of Science, Cochrane CENTRAL and Google Scholar databases. Databases were searched since their inception. The full search string is shown in Supplemental Figure S1. Studies were eligible for inclusion if they discussed tests applied to patients with a suspected myocardial contusion after blunt thoracic trauma. If no ‘myocardial contusion’ and ‘non-myocardial contusion’ group were mentioned, or if the diagnostic test that differentiated between the two groups was not known, the study was excluded. Studies on pediatric patients, animal studies, non-English studies, systematic reviews, meta-analyses, descriptive studies, and case reports were also excluded.

Literature selection and data extraction were done by two authors (DJTVS and EAD) independently. Any disagreement was resolved by consensus. First, titles and abstracts of all manuscript were reviewed for eligibility. Next, the full text of all remaining studies was screened for eligibility. For all included studies, the reference list was reviewed for identifying studies that were missed during the selection process.

Risk of bias assessment and applicability concerns for each study was carried out using the QUADAS-2 tool. For each study, the following data were extracted: author, publication year, study design, study population size, mean age, number of patients diagnosed with myocardial contusion, and the number of patients with ischemic heart disease or acute myocardial infarction. For echocardiography, electrocardiography, and cardiac biomarkers, the number of true and false positives and negatives were extracted for each study. In addition, the reference test and cut-off values for cardiac biomarkers used for identifying the myocardial contusion group were recorded.

Sensitivity and specificity were calculated using Review Manager (RevMan version 5.3, Copenhagen; The Nordic Cochrane Center, The Cochrane Collaboration, 2014). Subsequent meta-analysis was done using Meta-analysis of Diagnostic and Screening Tests (Meta-DiSc) [40]. Sensitivity and specificity were pooled across all studies, and a summary receiver operating curve was made. For each different diagnostic test, sensitivity and specificity are reported as percentage with 95% confidence interval. The area under the receiver operating curve (AUC) is reported with its standard error.

## Results

### Retrospective cohort study

**Fig. 1:**
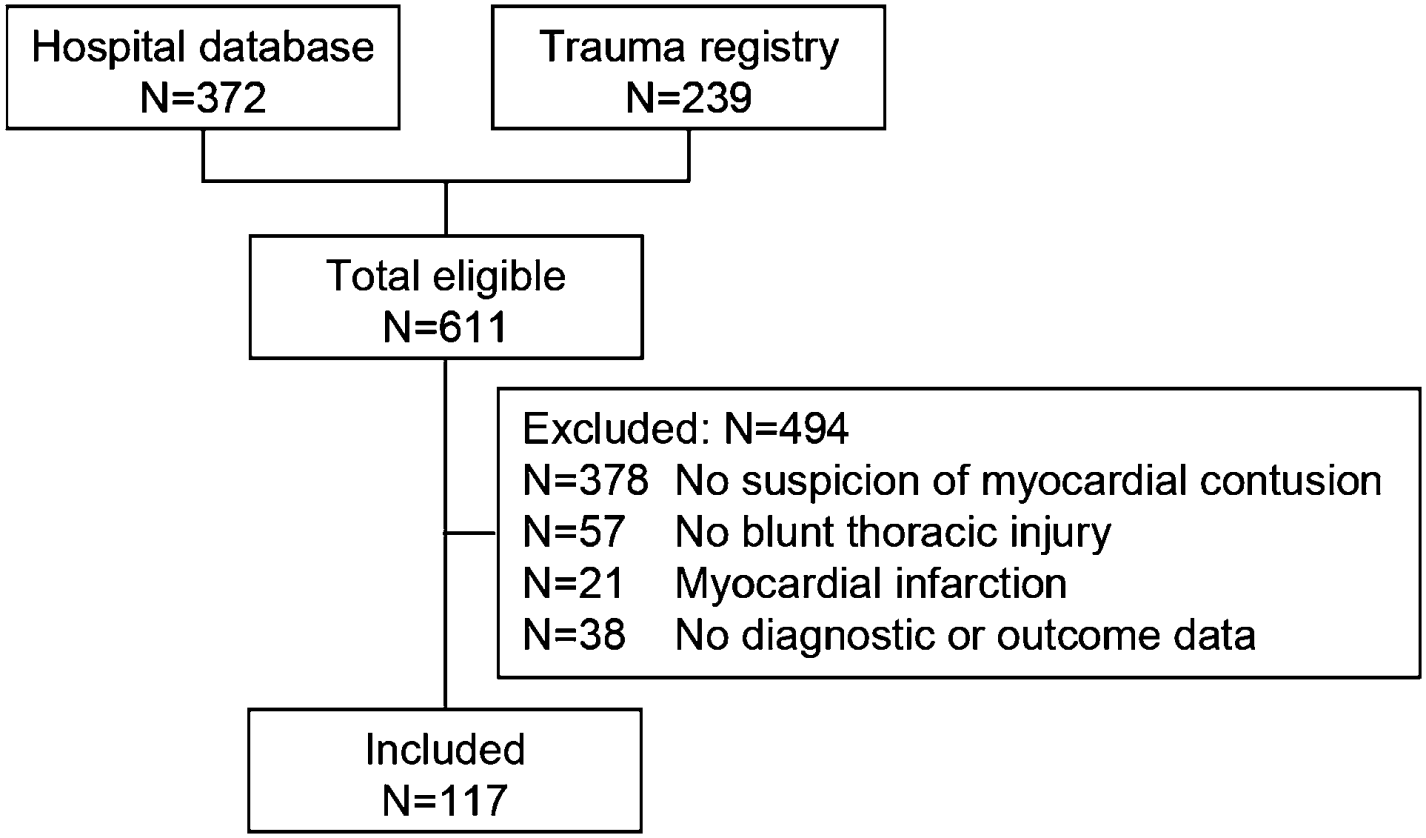
Flowchart of retrospective cohort study

A total of 611 patients were identified by searching the hospital database (*n* = 372) and the trauma registry (*n* = 239; Fig. 1). After studying the electronic medical records, 494 patients were excluded: 378 patients had not been suspected for myocardial contusion, 57 had not sustained blunt thoracic trauma, 21 had a confirmed myocardial infarction, and for 38 patients, diagnostic or outcome data were not available. The remaining 117 patients were admitted with a suspicion of myocardial contusion. Forty-four were diagnosed with a myocardial contusion and 73 were not. The study population had a median age of 43 (P_25_–P_75_ 31–62) years (Table 1), 83 (71%) patients were male and 16 (14%) had a known unrelated (cardiopulmonary) disorder. These patient characteristics at presentation did not differ between patients with myocardial contusion and those without myocardial contusion, nor did kidney function. Three patients used either citalopram, paroxetine, or methadone.

**Table 1:** Patient and injury characteristics of patients with versus without myocardial contusion

Parameter	Overall (*n* = 117)	Myocardial contusion (*n* = 44)	No myocardial contusion (*n* = 73)	*p* value
Patient characteristics				
Male gender	83 (71%)	34 (77%)	49 (67%)	0.296
Age	43 (31–62)	39 (23–64)	46 (34–61)	0.518
Comorbidity	16 (14%)	5 (11%)	11 (15%)	0.782
Cardiac	14 (12%)	4 (9%)	10 (14%)	ND
Pulmonary	3 (3%)	2 (5%)	1 (1%)	ND
Kidney transplant	1 (1%)	1 (2%)	0 (0%)	ND
Medication^a^	3 (3%)	0 (0%)	1 (4%)	0.290
Citalopram	1 (1%)	0 (0%)	1 (1%)	ND
Paroxetine	1 (1%)	0 (0%)	1 (1%)	ND
Methadone	1 (1%)	0 (0%)	1 (1%)	ND
eGFR at admission (mL/minute)	90 (75–105)	89 (68–107)	90 (79–104)	0.566
Thoracic injuries				
Thoracic injury	94 (80%)	40 (91%)	54 (74%)	0.031
Sternum fracture	67 (57%)	23 (52%)	44 (60%)	0.443
Rib fracture	61 (52%)	30 (68%)	31 (42%)	0.008
Pneumothorax	40 (34%)	19 (43%)	21 (29%)	0.159
Pulmonary contusion	36 (31%)	19 (43%)	17 (23%)	0.038
Hemothorax	18 (15%)	10 (23%)	8 (11%)	0.114
Flail chest	6 (5%)	2 (5%)	4 (5%)	1.000
Aorta rupture	2 (2%)	2 (5%)	0 (0%)	ND
Valve abnormalities	1 (1%)	1 (2%)	0 (0%)	ND

Data are shown as *n* (%) or median (P_25_–P_75_)

*eGFR* estimated glomerular filtration rate, *ND* not determined

^a^None of the patients had used amiodarone, haloperidol, flecainide, sotalol, macrolide antibiotics, cotrimoxazole, amitriptyline, bupropion, fluoxetine, sertraline, venlafaxine, domperidone, or ondansetron

The rate of non-cardiac thoracic injuries was higher in patients with a myocardial contusion (*n* = 40; 91%) than in those who were not diagnosed with a myocardial contusion (*n* = 54; 74%; *p* = 0.031). The four most common injuries were a sternum fracture, rib fracture(s), pneumothorax, and pulmonary contusion. Rib fractures occurred more often in patients with a myocardial contusion than patients without (68% versus 42%; *p* = 0.008). The same was true for pulmonary contusion (43% versus 23%; p = 0.038). Rates of all other thoracic injuries were similar in both groups.

Details of patient history could be found for 80% of patients; 39 (41%) patients had chest pain, 4 (4%) had dyspnea, 1 (1%) had palpitations, and none had cardiac rubbing (Table 2). None of these differed between the two groups. Results of physical examination were available for all 117 patients. Patients with a myocardial contusion had a higher median heart rate (97 bpm; P_25_–P_75_ 82–114 versus 80; P_25_–P_75_ 72–92; *p* < 0.001), a lower median mean arterial pressure (MAP) (94 mmHg; *P*_25_–*P*_75_ 73–107 versus 100 mmHg; *P*_25_–*P*_75_ 94–110; *p* = 0.037), and a lower median Glasgow Coma Scale (15; *P*_25_–*P*_75_ 5–15 versus 15; *P*_25_–*P*_75_ 15–15; *p* = 0.001).

**Table 2:** Results from patient history and physical examination of patients with versus without myocardial contusion

Parameter	Overall (*n* = 117)	Myocardial contusion (*n* = 44)	No myocardial contusion (*n* = 73)	*p* value
Diagnostic item at presentation				
Patient history recorded	94 (80%)	30 (68%)	64 (88%)	0.016
Chest pain	39/94 (41%)	9/30 (30%)	30/64 (47%)	0.178
Dyspnea	4/94 (4%)	3/30 (10%)	1/64 (2%)	0.094
Palpitations	1/94 (1%)	1/30 (3%)	0/64 (0%)	ND
Physical examination recorded	117 (100%)	44 (100%)	73 (100%)	ND
Cardiac murmurs	3/117 (3%)	2/44 (5%)	1/73 (1%)	0.555
Heart rate (bpm)	85 (74–100)	97 (82–114)	80 (72–92)	< 0.001
MAP (mmHg)	80 (70–89)	94 (73–107)	100 (94–110)	0.024
Chest X-ray abnormality	0/114 (0%)	0/42 (0%)	0/72 (0%)	ND
Chest CT scan abnormality	6/83 (7%)	1/33 (3%)	5/50 (10%)	0.395
ECG abnormality	10/96 (10%)	5/35 (14%)	5/61 (8%)	0.489
TTE abnormality	1/35 (3%)	1/10 (10%)	0/25 (0%)	0.286
TnT measured	51 (44%)	22 (50%)	29 (40%)	0.337
TnT (ng/L)	0 (0–40)	55 (0–283)	0 (0–0)	< 0.001
TnT elevated	13/51 (25%)	13/22 (59%)	0/29 (0%)	< 0.001
Hs-TnT measured	61 (52%)	22 (50%)	39 (53%)	0.849
Hs-Tnt (ng/L)	10 (5–25)	28 (19–57)	5 (3–11)	< 0.001
Hs-TnT elevated	23/61 (38%)	17/22 (77%)	6/39 (15%)	< 0.001
CK-MB measured	108 (92%)	44 (100%)	64 (88%)	0.013
CK-MB (µg/L)	8 (4–17)	15 (7–28)	5 (3–10)	< 0.001
CK-MB elevated	57/108 (53%)	33/44 (75%)	24/75 (38%)	< 0.001
Diagnostic item at follow-up				
Observation	111 (95%)	43 (98%)	68 (93%)	0.407
Telemonitoring	83 (71%)	40 (91%)	43 (59%)	< 0.001
Telemonitoring (days)	2 (2–7)	4 (2–10)	2 (2–3)	0.057
ECG abnormality	3/37 (8%)	1/17 (6%)	2/20 (10%)	1.000
Time to repeated lab tests (h)	4 (3–6)	4 (3–7)	4 (3–6)	0.833
TnT measured	48 (41%)	21 (48%)	27 (37%)	0.332
TnT (ng/L)	0 (0–148)	180 (55–1295)	0 (0–0)	< 0.001
TnT elevated	20 (42%)	18 (86%)	2 (7%)	< 0.001
Hs-TnT measured	52 (44%)	21 (48%)	31 (42%)	0.701
Hs-Tnt (ng/L)	12 (6–56)	56 (31–127)	8 (5–12)	< 0.001
Hs-TnT elevated	25 (48%)	19 (90%)	6 (19%)	< 0.001
CK-MB measured	95 (81%)	40 (91%)	55 (75%)	0.050
CK-MB (µg/L)	14 (6–29)	23 (11–43)	9 (4–18)	< 0.001
CK-MB elevated	68 (72%)	37 (93%)	31 (56%)	< 0.001

Data are shown for the diagnostic modalities at presentation to hospital

Troponin I is not shown, as it was measured in one patient (who had myocardial contusion) at presentation, and in none at follow-up

Data are shown as *n* (%) or as median (P_25_–P_75_)

*Bpm* beats per minute, *CI* confidence interval, *CK-MB* creatine kinase, muscle and brain isoenzyme, *CT* computed tomography, *ECG* electrocardiography, *Hs-TnT* high sensitive troponin T, *MAP* mean arterial pressure, *ND* not determined, *TTE* transthoracic echocardiography

Chest CT scan, electrocardiography, and transthoracic echocardiography made at hospital presentation were indicative of myocardial contusion in only one patient who showed retrosternal hematoma (Table 2). Five patients without myocardial contusion also showed retrosternal hematoma on the CT scan. Chest X-ray was unremarkable in all patients. On the other hand, levels of cardiac biomarkers TnT, hs-TnT, and CK-MB were consistently higher in patients with a myocardial contusion than in patients without.

Overall, 111 patients with a suspected myocardial contusion were admitted for observation. Telemonitoring was done in 91% of patients with a myocardial contusion versus 59% of patients without myocardial contusion (*p* < 0.001). Abnormalities suggestive of myocardial contusion at the electrocardiography made during admission were shown only in one patient with a myocardial contusion. Levels of cardiac biomarkers remained higher at follow-up in patients with a myocardial contusion than in patients without, as was the case in the initial screening.

**Fig. 2:**
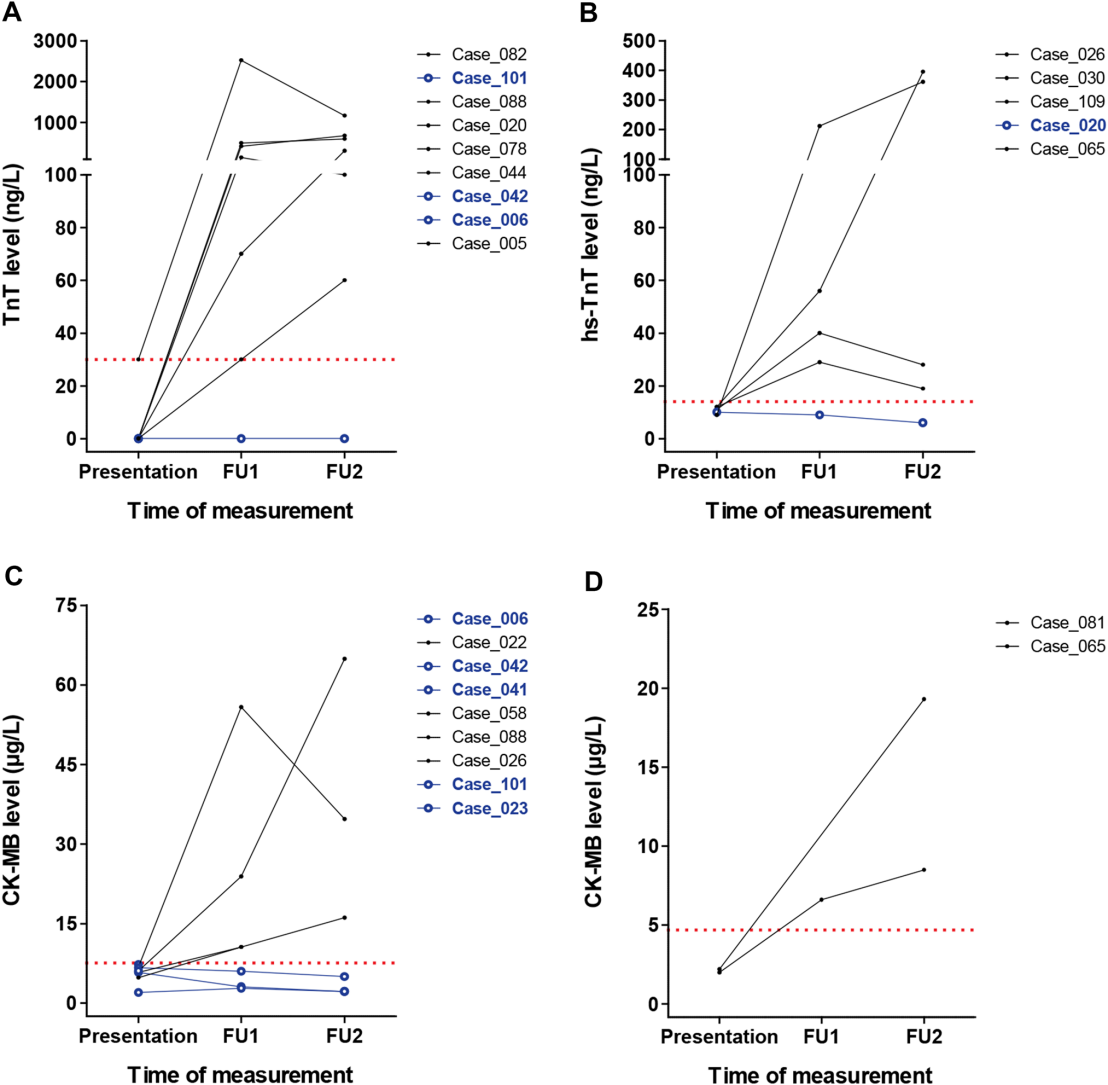
Change in cardiac biomarkers over time in patients with myocardial contusion who had their first measurement below the threshold value. Results are shown for **a** TnT, **b** hs-TnT, and CK-MB in **c** males and **d** females. Blue lines indicate patients with measurements that remain below the threshold during follow-up. Red dotted lines show the threshold above which the cardiac biomarker is considered elevated. For CK-MB, males and females have a different threshold

The diagnostic properties for myocardial contusion of all diagnostic tests evaluated performed at presentation and during follow-up are shown in Table 3. Chest X-ray, chest CT scan, electrocardiography, and echocardiography all had a poor sensitivity (< 15%) but a good specificity (≥ 90%). Diagnostic performance is much better for the cardiac biomarkers. Sensitivity ranged from 59% for TnT to 77% for hs-TnT, measured at presentation. Repeated cardiac biomarkers even had a sensitivity of 86% for TnT and 93% for hs-TnT. Specificity for measurements at presentation and follow-up was excellent for TnT (100% and 93%) and good for hs-TnT (85% and 81%), but moderate to poor for CK-MB (63% and 44%). Plot of the cardiac biomarkers over time for patients with myocardial contusion that did not have elevated marker expression at presentation is shown in Fig. 2. This shows that six out of nine patients with normal TnT values at presentation had elevated TnT expression at both repeated measurements (Fig. 2a). Likewise, four out of five patients with normal hs-TnT expression at presentation had elevated levels at follow-up (Fig. 2b). For CK-MB, four out of seven males and both females had elevated CK-MB expression, following normal expression at presentation (Fig. 2c, d).

**Table 3:** Diagnostic properties of all diagnostic tests performed

Test	*n*	Prevalence (95% CI)	Sensitivity (95% CI)	Specificity (95% CI)
Chest X-ray	114	37% (28–46%)	0% (0–0%)	100% (95–100%)
Chest CT scan	83	40% (29–51%)	3% (0–16%)	90% (78–97%)
ECG at presentation	96	36% (27–47%)	14% (5–30%)	92% (82–97%)
ECG at follow-up^a^	37	46% (29–63%)	6% (0–29%)	90% (68–99%)
TTE at presentation	35	29% (14–46%)	10% (0–45%)	100% (86–100%)
Cardiac biomarkers				
TnT at presentation	51	43% (29–58%)	59% (36–79%)	100% (88–100%)
TnT at follow-up^a^	48	29% (29–59%)	86% (64–97%)	93% (76–99%)
Hs-TnT at presentation	61	36% (24–49%)	77% (55–92%)	85% (69–94%)
Hs-TnT at follow-up^a^	52	40% (27–55%)	90% (70–99%)	81% (63–93%)
CK-MB at presentation	108	41% (31–51%)	75% (60–87%)	63% (50–74%)
CK-MB at follow-up^a^	95	42% (32–53%)	93% (80–98%)	44% (30–57%)

Data for sensitivity and specificity are shown as % (95% CI)

Troponin I is not shown, as it was measured in one patient (who had myocardial contusion) at presentation, and in none at follow-up

*CI* confidence interval, *CK-MB* creatine kinase, muscle and brain isoenzyme, *CT* computed tomography, *ECG* electrocardiography, *Hs-TnT* high-sensitive troponin T, *TnT* troponin T, *TTE* transthoracic echocardiography

^a^Median time to follow-up was 4 (2–10) days in patients with a myocardial contusion and 2 (2–3) days in patients without myocardial contusion for telemetric observation, and 4 (3–7) h in patients with a myocardial contusion and 4 (3–6) h in patients without myocardial contusion for laboratory tests

Thirty-three cardiac adverse events had developed in 18 patients (Table 4). Patients with myocardial contusion had a larger rate of cardiac adverse events (*n* = 15; 34%) than controls (*n* = 3; 4%; *p* < 0.001). The most common adverse event was arrhythmia, which was seen in 13 patients with a myocardial contusion (versus two in patients without myocardial contusion). Eight of these 13 patients with a myocardial contusion had atrial fibrillation or flutter. Rib fractures occurred more often in patients who developed an adverse event: 12 out of 18 (67%) patients with an adverse event had fractured ribs, versus 49 out of 99 (49%) in patients who remained free of adverse events. Seven patients died, four patients with a myocardial contusion and three patients without myocardial contusion. Of the patients with myocardial contusion, one patient died of cardiogenic shock due to a known mitral valve dysfunction; in two other patients, treatment was stopped due to infaust neurological prognosis after trauma. In the fourth patient, no cause of death was noted and autopsy was not performed. One patient without myocardial contusion died of pulmonary embolism, the other two due to infaust neurological prognosis after trauma.

**Table 4:** Adverse events and mortality of patients with versus without myocardial contusion

Parameter	Overall (*n* = 117)	Myocardial contusion (*n* = 44)	No myocardial contusion (*n* = 73)	*p* value
Adverse events	18 (15%)	15 (34%)	3 (4%)^a^	< 0.001
Arrhythmias	14 (12%)	13 (30%)	1 (1%)^a^	< 0.001
Atrial fibrillation	7 (6%)	7 (16%)	0 (0%)	ND
PVC	2 (2%)	2 (5%)	0 (0%)	ND
Atrial flutter	1 (1%)	1 (2%)	0 (0%)	ND
Hypotension	8 (7%)	6 (14%)	2 (3%)	0.051
Cardiogenic shock	1 (1%)	1 (2%)	0 (0%)	0.376
Mortality	7 (6%)	4 (9%)	3 (4%)	0.423

Data are shown as *n* (%)

*ND* not determined, *PVC* premature ventricular complexes

^a^This is excluding one patient for whom an electrocardiography before trauma already showed atrial fibrillation

### Literature review

**Fig. 3:**
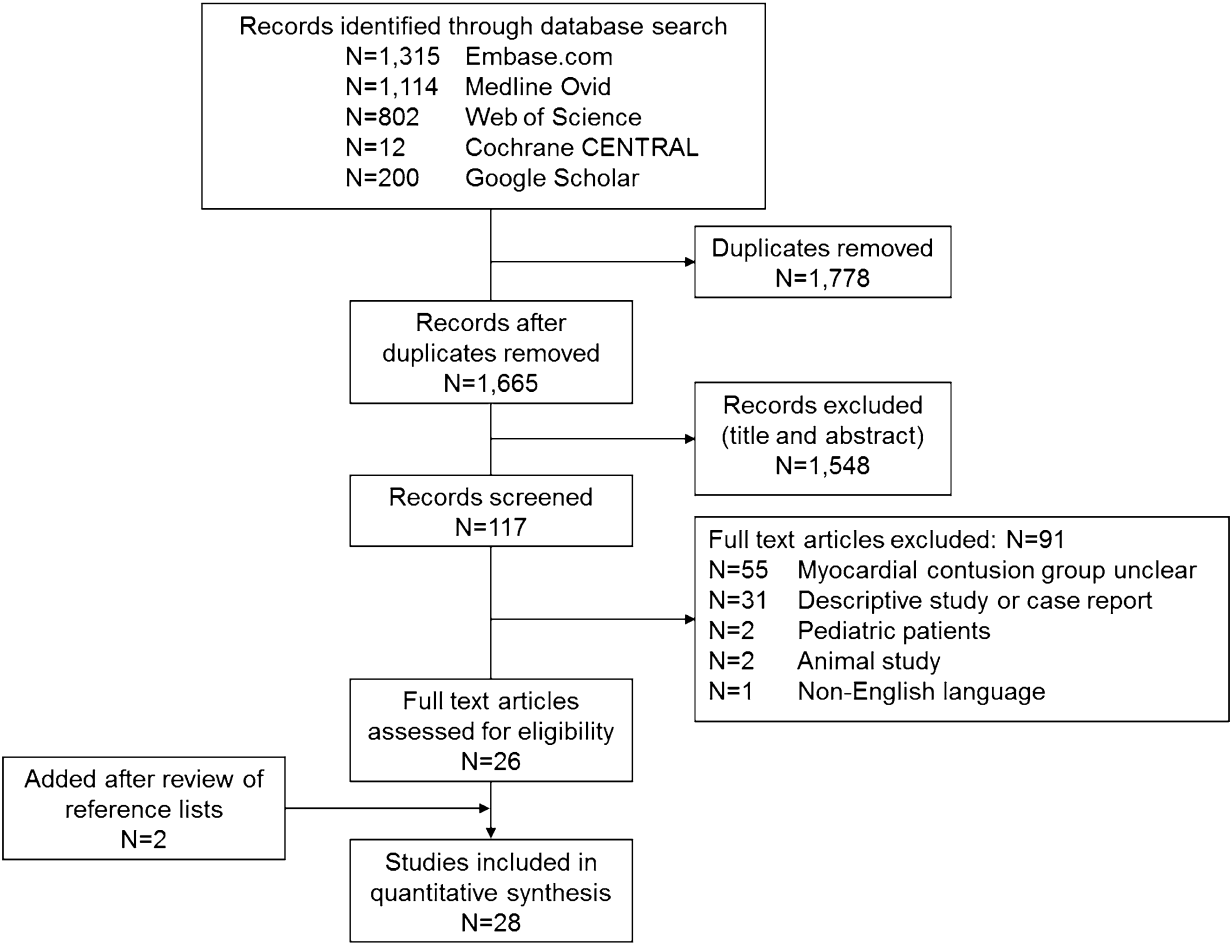
Flowchart of literature search

The search strategy resulted in 3443 records, of which 1665 remained after removing duplicates (Fig. 3). After applying inclusion criteria to title and abstract, 117 records remained. Of these, 91 had to be excluded based upon the exclusion criteria. Review of reference lists of the 26 included manuscript yielded 2 studies that were missed in the selection process. Details on the 28 included manuscripts are shown in Supplemental Table S1 [2, 3, 8, 14, 21–23, 35–37, 41–58]. Twenty-four manuscripts were prospective studies. The total sample size of all studies was 7242 patients, of whom 1,048 (14.5%) were labeled as having a myocardial contusion. The diagnostic criteria for myocardial contusion varied across studies, but mostly elevated cardiac biomarkers or abnormalities seen on electrocardiography or echocardiography, or a combination of both.

**Fig. 4:**
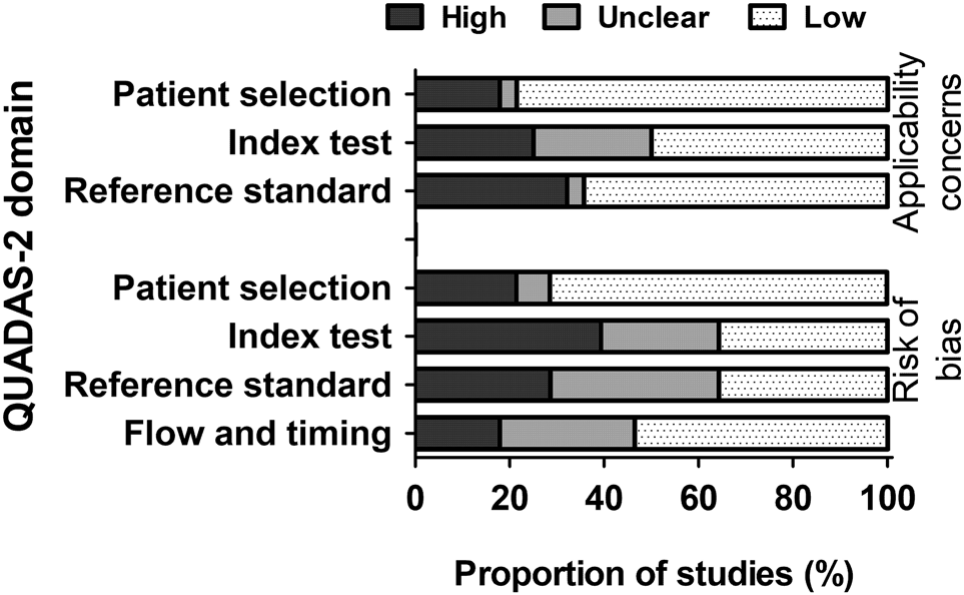
Risk of bias and applicability concerns graph. Authors’ judgements about each domain presented as percentages across included studies

The risk of bias was judged as low for most studies in the domains of patient selection (*n* = 20), conduct or interpretation of the index test (*n* = 10), the reference standard (*n* = 10), and patient flow and timing (*n* = 15; Fig. 4). Applicability concerns were judged as low risk of bias for most studies in the domains of patient selection (*n* = 22), index test (*n* = 14), and reference standard (*n* = 18).

Sensitivity and specificity of all diagnostic tests used per study are shown in Supplemental Figure S2, the pooled values are shown in Table 5. The enrolled studies did not allow pooling of data for hs-TnT. The heterogeneity for all diagnostic tests is large, as expressed by a significant Chi-square test and *I*^2^ value > 50%. This warrants careful interpretation of the pooled sensitivity and specificity. The pooled sensitivity of electrocardiography, troponin I, and CK-MB for identifying myocardial contusion varied between 62 and 71%. Values were 45% for echocardiography and even 38% for troponin T. The pooled specificity was generally better and ranged from 63% for CK-MB to 85% for troponin T and 88% for echocardiography. The area under the summary receiver operating curve ranged from 0.71 for CK-MB to 0.91 for echocardiography.

**Table 5:** Pooled sensitivity and specificity of all diagnostic tests for identifying myocardial contusion for all studies identified in the systematic literature review

Test	Sensitivity			Specificity			AUC
	Chi^2^ (*p*)	*I*^2^ (%)	Pooled % (95% CI)	Chi^2^ (*p*)	*I*^2^ (%)	Pooled % (95% CI)	
Electrocardiography	165 (< 0.001)	93	71% (66–75%)	262 (< 0.001)	96	75% (72–77%)	0.86 (0.04)
Echocardiography	43 (< 0.001)	84	45% (37–53%)	23 (< 0.001)	69	88% (83–92%)	0.91 (0.08)
Cardiac biomarkers							
Troponin T	26 (0.001)	88	38% (27–50%)	61 (< 0.001)	95	85% (83–87%)	0.88 (0.17)
Troponin I	33 (< 0.001)	79	62% (53–69%)	127 (< 0.001)	95	76% (73–80%)	0.80 (0.07)
CK-MB	61 (< 0.001)	86	66% (60–72%)	291 (< 0.001)	97	63% (59–67%)	0.71 (0.06)

## Discussion

If a patient is presented to the hospital after having sustained blunt thoracic trauma, a myocardial contusion should be considered. Results of the current study show that the highest sensitivity and specificity are achieved when electrocardiography is combined with measuring cardiac biomarkers. This combination is best used for ruling in the disorder. Echocardiography is valuable for visual inspection of the heart. Chest X-ray and chest CT scan are valuable for identifying thoracic injuries and intra-thoracic bleeding, but are not useful for identifying myocardial contusion.

The gold standard for identifying myocardial contusion is pathologic evaluation of the cardiac tissue post-mortem. Necrosis of cardiac myocytes is the only confirmative proof.

Since microscopic evaluation is not possible in a clinical setting, diagnostic tools such as electrocardiography, echocardiography, and measurement of cardiac biomarkers are needed.

**Fig. 5:**
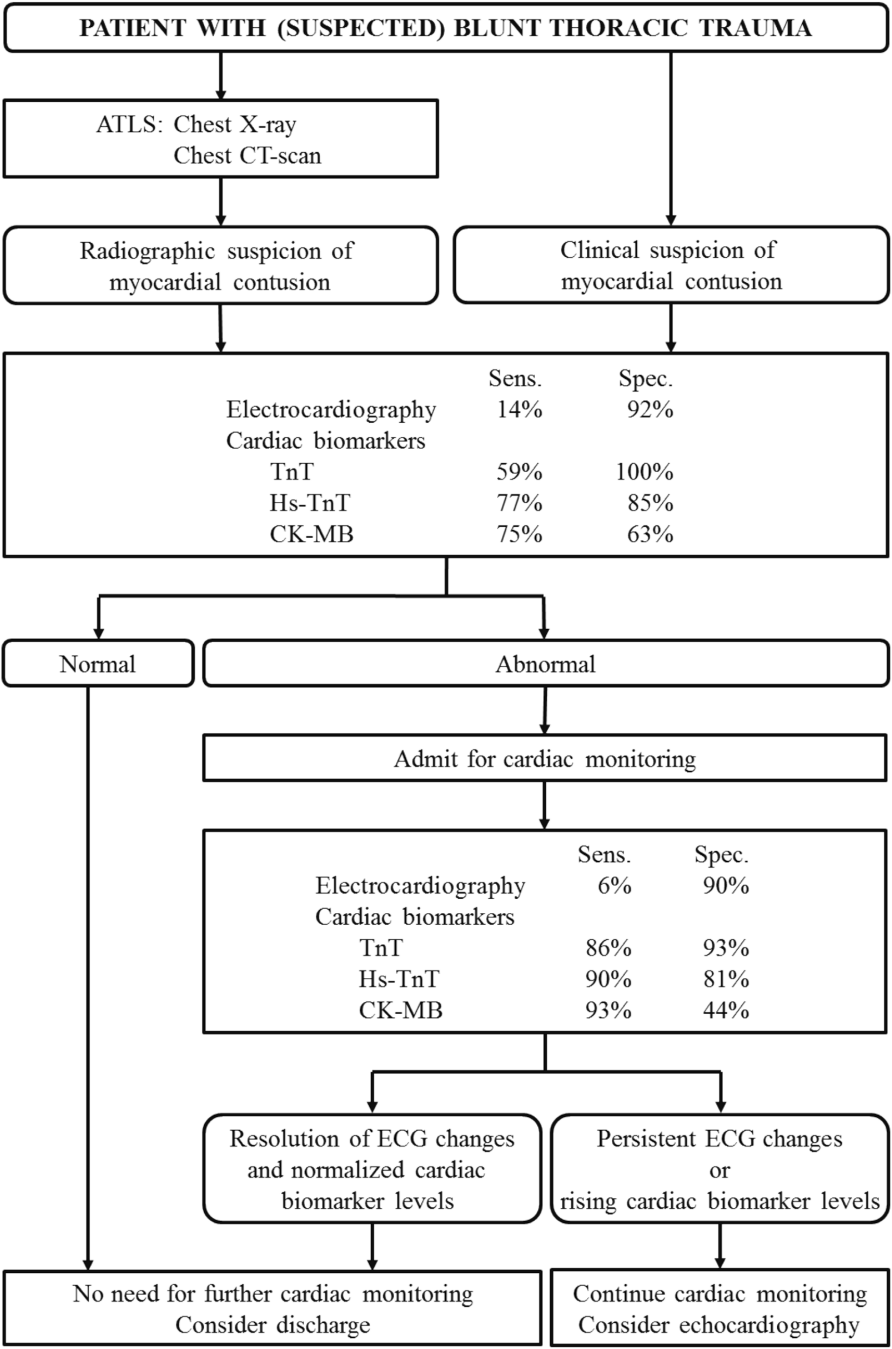
Diagnostic algorithm for patients with blunt thoracic injuries with suspected myocardial contusion

The current meta-analysis supports the diagnostic algorithms that were recently published [59–61]. Based on our data, we suggest a diagnostic workup as depicted in Fig. 5. In line with the Advanced Trauma Life Support guidelines, a chest X-ray or CT scan is indicated for patients presenting to the hospital after blunt thoracic trauma [62]. These do not provide any information on cardiac involvement per se; however, these tests are required to rule out associated thoracic injuries which could serve as an alert to the possibility of a cardiac injury (e.g., pulmonary contusion or fractures of the rib or sternum) [63].

Upon presentation to the hospital, electrocardiography and measurement of cardiac biomarkers should routinely be done in patients who sustained blunt thoracic trauma. An electrocardiogram doubtful of myocardial contusion may reveal cardiac arrhythmias indicative for intraventricular conduction disorders, persistent atrial fibrillation, premature ventricular complexes, sinus tachycardia, a new bundle branch block, or ST depressions or elevations [9, 64–68]. Despite the fact that electrocardiography has low sensitivity and specificity when used alone [45], patients with an abnormal electrocardiogram develop more significant complications that require treatment [24]. The difficulty is to determine if the abnormality on an electrocardiogram is a primary event (e.g., an acute coronary syndrome that preceded trauma), a direct result of a cardiac injury, or a problem caused by the physiological stress of severe chest trauma.

Electrocardiography has consistently proven to be the best single overall predictor of blunt cardiac trauma [69]. However, electrocardiography alone is insufficient to completely exclude the diagnosis, and in a few rare cases has even missed significant blunt chest injuries [23, 47]. On the other hand, in our series, two control patients showed cardiac arrhythmias. Therefore, cardiac biomarkers (troponins T and I and CK-MB) should also be measured. Although the usefulness of cardiac biomarkers is unclear [36, 47], elevated troponin levels have been associated with increased mortality in patients with blunt thoracic trauma [42]. Although sensitivity and specificity of cardiac biomarkers vary largely across studies on myocardial contusion [2, 3, 8, 14, 21–23, 35–37, 41–58], our results show that sensitivity and specificity of the cardiac biomarkers is higher than that of echocardiography. Since other cardiac conditions may also result in cardiac biomarker elevation, cardiac biomarkers should be combined with either electrocardiography or echocardiography. Elevation of cardiac biomarkers can also be the result of hypovolemic shock, which is commonly seen in patients with significant chest trauma.

Electrocardiogram abnormalities or rising troponin values should prompt further evaluation using transthoracic echocardiography (TTE). This may reveal left or right ventricular systolic dysfunction, pericardial effusion with suspected tamponade, ventricular septal defect, or possible trauma-induced valvular abnormalities [70].

Results from the electrocardiography (and echocardiography) and cardiac biomarkers support the subsequent diagnostic necessities and monitoring. Patients with abnormalities on the electrocardiography (or echocardiography) that are not explained by (non-)STEMI or by cardiac diseases that were already present pre-trauma, should be considered suspect for myocardial contusion. If cardiac biomarkers are also elevated, measurement of these markers should be repeated after 3 h. In addition, telemonitoring is required for early knowledge of possible development of life-threatening arrhythmias or other complications. The patient data in this study confirm the relevance of this as 13 patients with myocardial contusion developed some form or arrhythmia over time. Cardiac monitoring should last at least 24–48 h because life-threatening ventricular arrhythmias, cardiac failure due to valve damage, cardiac tamponade due to a wall rupture, or acute coronary syndrome due to coronary artery dissection may develop within this period [35, 60, 67, 71, 72].

If cardiac biomarkers are not elevated in patients with electrocardiography abnormalities, the biomarkers should be measured again after 3 h, as they may become positive. If the cardiac biomarkers remain negative and no other clinical suspicion of myocardial contusion has emerged, the diagnosis can be rejected. Emerging clinical suspicion or elevated markers at follow-up, on the other hand, supports the presence of myocardial contusion. Telemonitoring is indicated in those patients.

If both cardiac biomarkers are not elevated and the electrocardiography shows no abnormalities, myocardial contusion is unlikely. It was shown previously that patients with a normal electrocardiogram in conjunction with normal levels of troponin can be safely discharged home [60, 67, 68]. However, our patient data show that clinical observation without telemonitoring may be needed for patients who have other traumatic thoracic diagnoses such as rib fractures.

The current study has several limitations. The most obvious limitations are the retrospective nature of the cohort study and the lack of a gold standard in both the cohort study and all studies in the literature review. Personal opinion of the treatment team may have led to false positive of false negative results for myocardial contusion. These differences in diagnostic criteria used, variation in biomarker cut off values, as well as differences in study population may have contributed to the heterogeneity across studies in the meta-analysis. This may have resulted in differences in sensitivity and specificity of the different diagnostic tests across the published as well as our cohort study. This heterogeneity also warrants careful interpretation of the pooled sensitivity and specificity.

## Conclusions

Data from the current study support that initial diagnostic workup in patients who presented to a hospital after blunt thoracic trauma should consist of electrocardiography and measurement of cardiac biomarkers. If the electrocardiography shows abnormalities indicative of myocardial contusion and/or cardiac biomarkers are elevated at presentation or become elevated within the subsequent 3 h, echocardiography and telemonitoring are indicated. If electrocardiography is normal and cardiac biomarkers remain negative, clinical observation may suffice. Further prospective studies are needed to refine the proposed diagnostic scheme.

## Electronic supplementary material

Below is the link to the electronic supplementary material.

Supplementary file1 (DOCX 158 kb)

Supplementary file2 (DOCX 45 kb)

## Abbreviations

TnT: Troponin T
TnI: Troponin I
CK-MB: Creatine kinase muscle/brain
(non-)STEMI: (Non)-ST elevation myocardial infarction
ECG: Electrocardiogram
SPSS: Statistical Package for the Social Sciences
RevMan: Review Manager
Meta-DiSc: Meta-analysis of Diagnostic and Screening Tests
AUC: Area under the receiver operating curve
MAP: Mean arterial pressure
TTE: Transthoracic echocardiography
