# Supplementary material for: Diagnostic approach for myocardial contusion: a retrospective evaluation of patient data and review of the literature
Source: Eur J Trauma Emerg Surg. 2020 Jan 25;47(4):1259–72. doi: 10.1007/s00068-020-01305-4 (PMC8321993; doi:10.1007/s00068-020-01305-4)
Supplement: Supplementary file 1 — Supplementary file1 (DOCX 158 kb) [file 68_2020_1305_MOESM1_ESM.docx]

**Supplemental Figure S1: Search string for systematic literature review**

***Medline Ovid***: (exp "Myocardial Contusions"/ OR (((heart OR cardiac OR myocard* OR cordis) ADJ6 (contusio* OR commotio* OR concussion*)) OR ((blunt* OR Nonpenetrat* OR Non-penetrat*) ADJ6 (heart OR cardiac*) ADJ6 (trauma* OR injur*))).ab,ti,kf.) NOT (exp animals/ NOT humans/) NOT ((letter OR news OR comment OR editorial OR congresses OR abstracts).pt.) AND english.la

***Medline Ovid***: (exp "Myocardial Contusions"/ OR (((heart OR cardiac OR myocard* OR cordis) ADJ6 (contusio* OR commotio* OR concussion*)) OR ((blunt* OR Nonpenetrat* OR Non-penetrat*) ADJ6 (heart OR cardiac*) ADJ6 (trauma* OR injur*))).ab,ti,kf.) NOT (exp animals/ NOT humans/) NOT ((letter OR news OR comment OR editorial OR congresses OR abstracts).pt.) AND english.la.

***Embase.com***: ('heart contusion'/exp OR 'commotio cordis'/de OR (((heart OR cardiac OR myocard* OR cordis) NEAR/6 (contusio* OR commotio* OR concussion*)) OR ((blunt* OR Nonpenetrat* OR Non-penetrat*) NEAR/6 (heart OR cardiac*) NEAR/6 (trauma* OR injur*))):ab,ti) NOT ([animals]/lim NOT [humans]/lim) NOT ([Conference Abstract]/lim OR [Letter]/lim OR [Note]/lim OR [Editorial]/lim) AND [english]/lim

***Cochrane CENTRAL***: ((((heart OR cardiac OR myocard* OR cordis) NEAR/6 (contusio* OR commotio* OR concussion*)) OR ((blunt* OR Nonpenetrat* OR Non-penetrat*) NEAR/6 (heart OR cardiac*) NEAR/6 (trauma* OR injur*))):ab,ti)

***Web of science***: TS=(((((heart OR cardiac OR myocard* OR cordis) NEAR/5 (contusio* OR commotio* OR concussion*)) OR ((blunt* OR Nonpenetrat* OR Non-penetrat*) NEAR/5 (heart OR cardiac*) NEAR/5 (trauma* OR injur*)))) ) AND DT=(article) AND LA=(english)

***Google scholar***: "heart|cardiac|myocardial contusion|concussion"|"commotio cordis"|"blunt|Nonpenetrating heart|cardiac trauma|injury|injuries" diagnosis|diagnostic

**Supplemental Figure S2: Sensitivity, specificity, and summary receiver operating curves (SROC) of electrocardiography, echocardiography, and laboratory tests for identifying myocardial contusion**

**A: Electrocardiography**

**
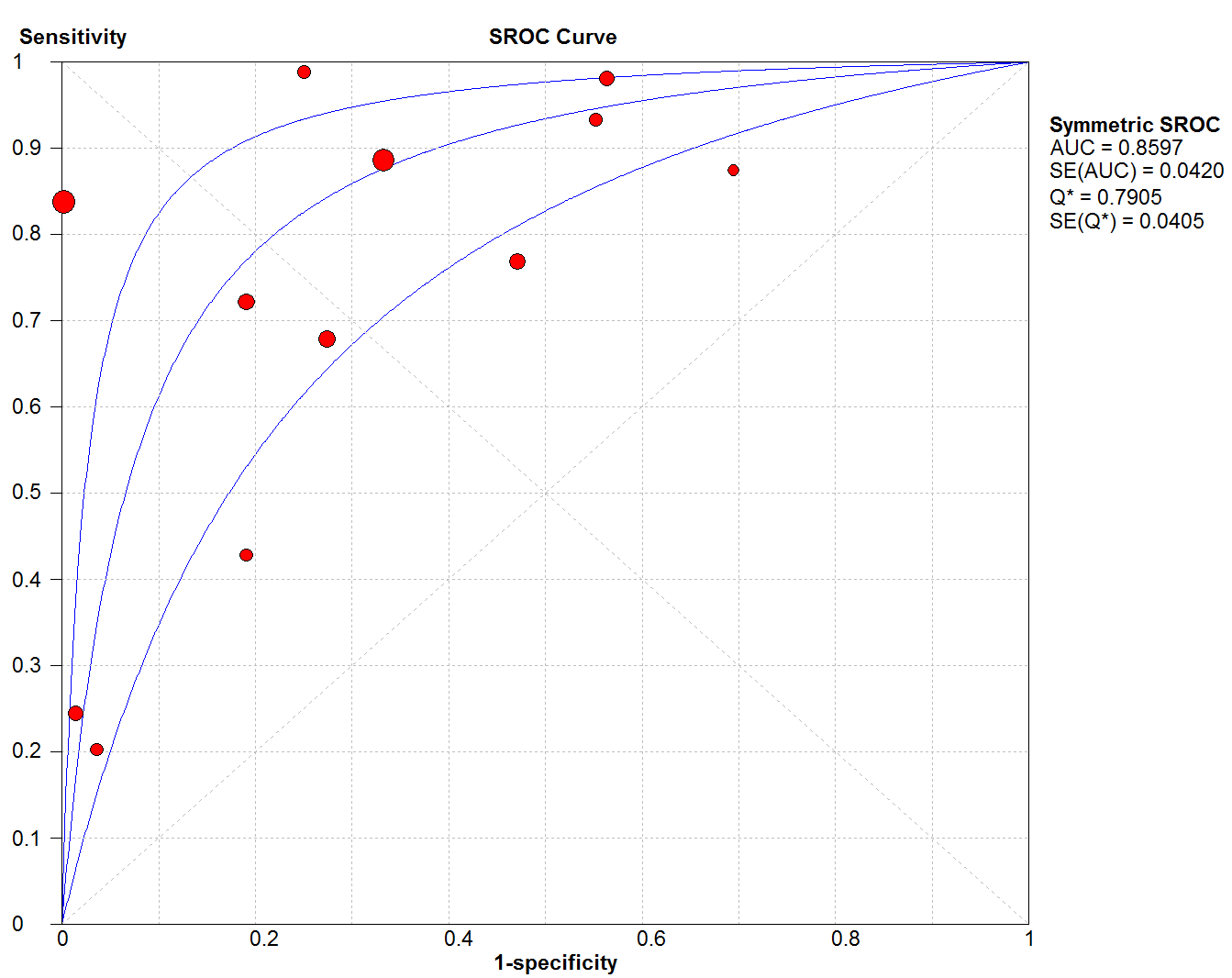
**

**B: Echocardiography**

**
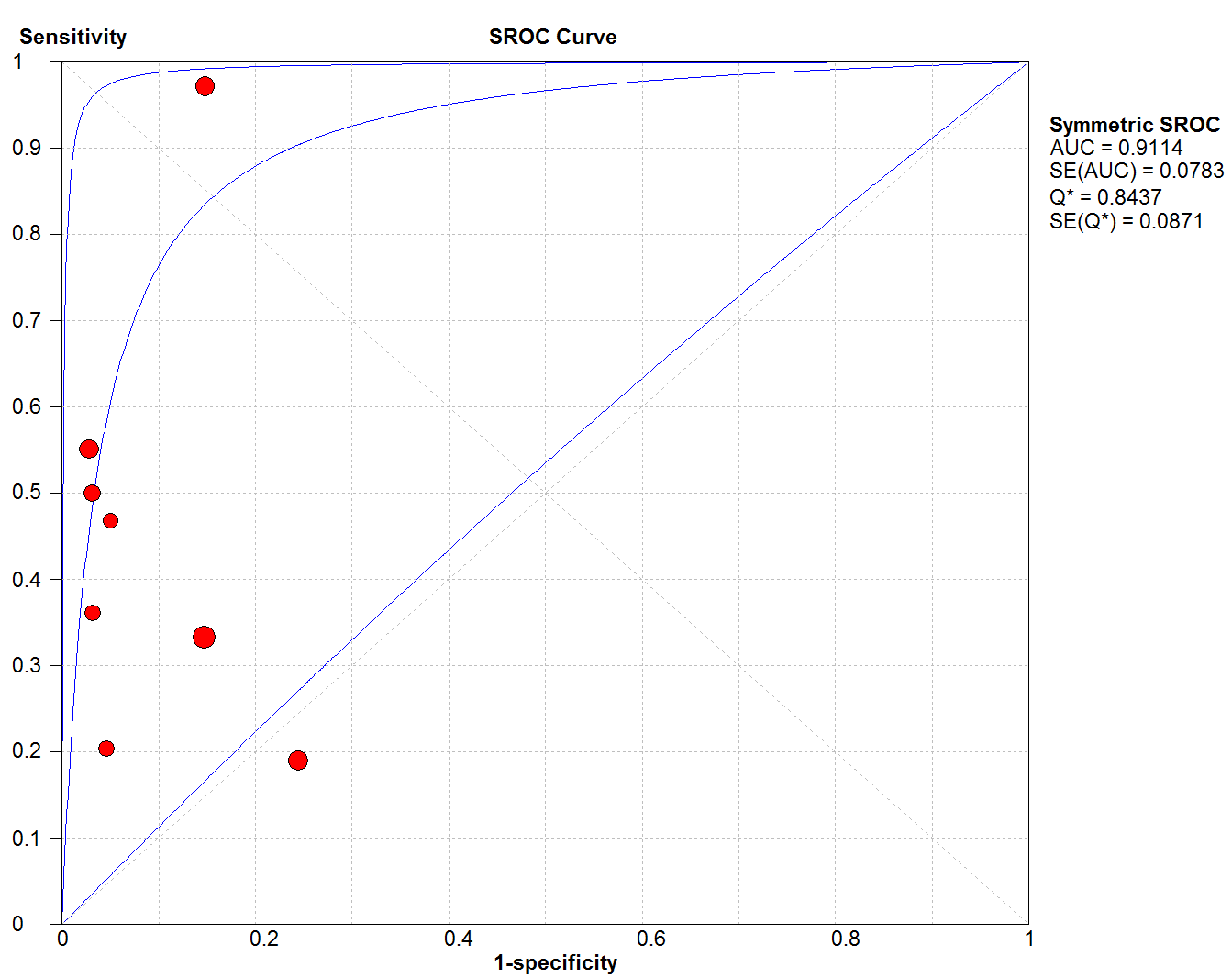
**

**C: Troponin T**

**
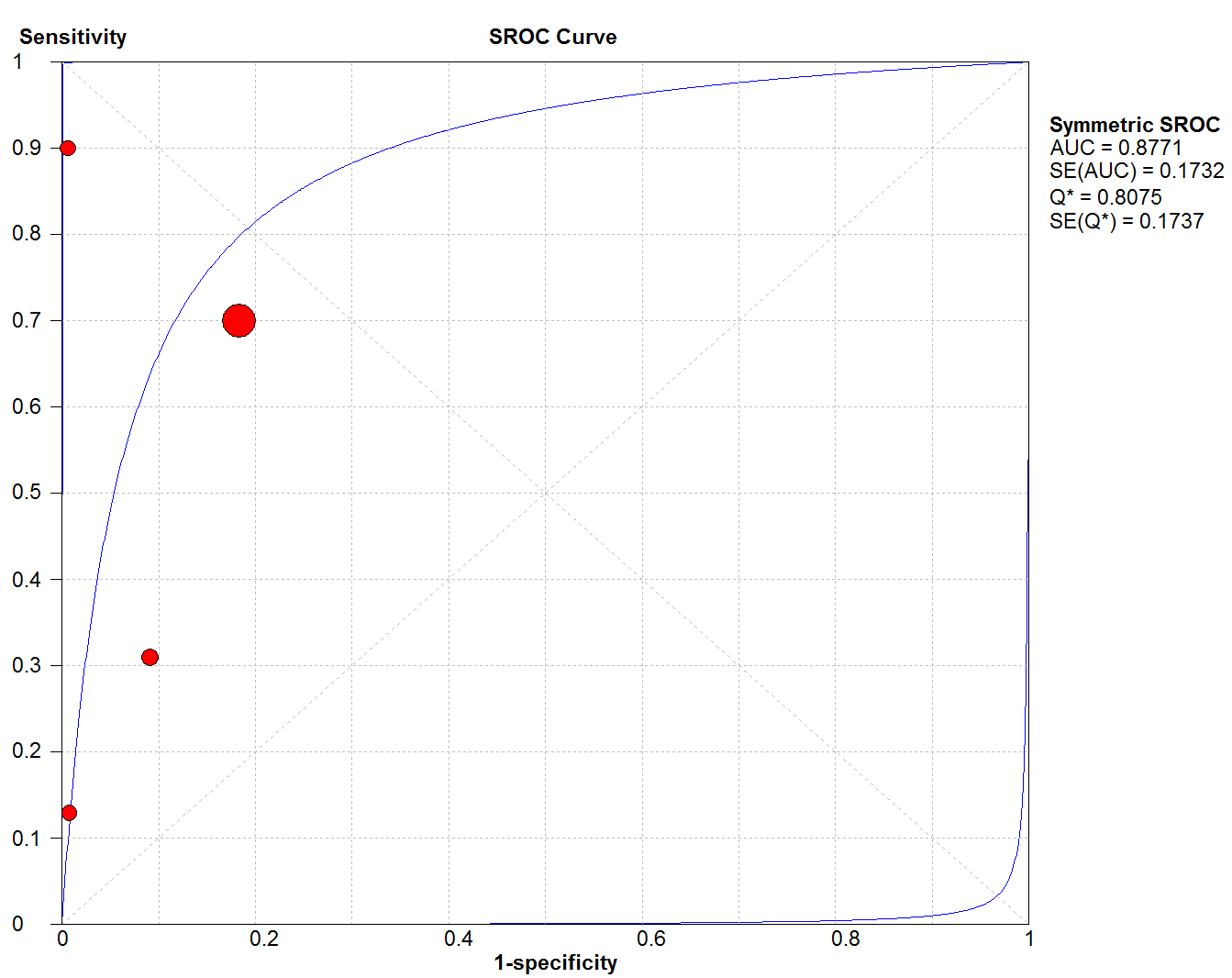
**

**D: Troponin I**

**
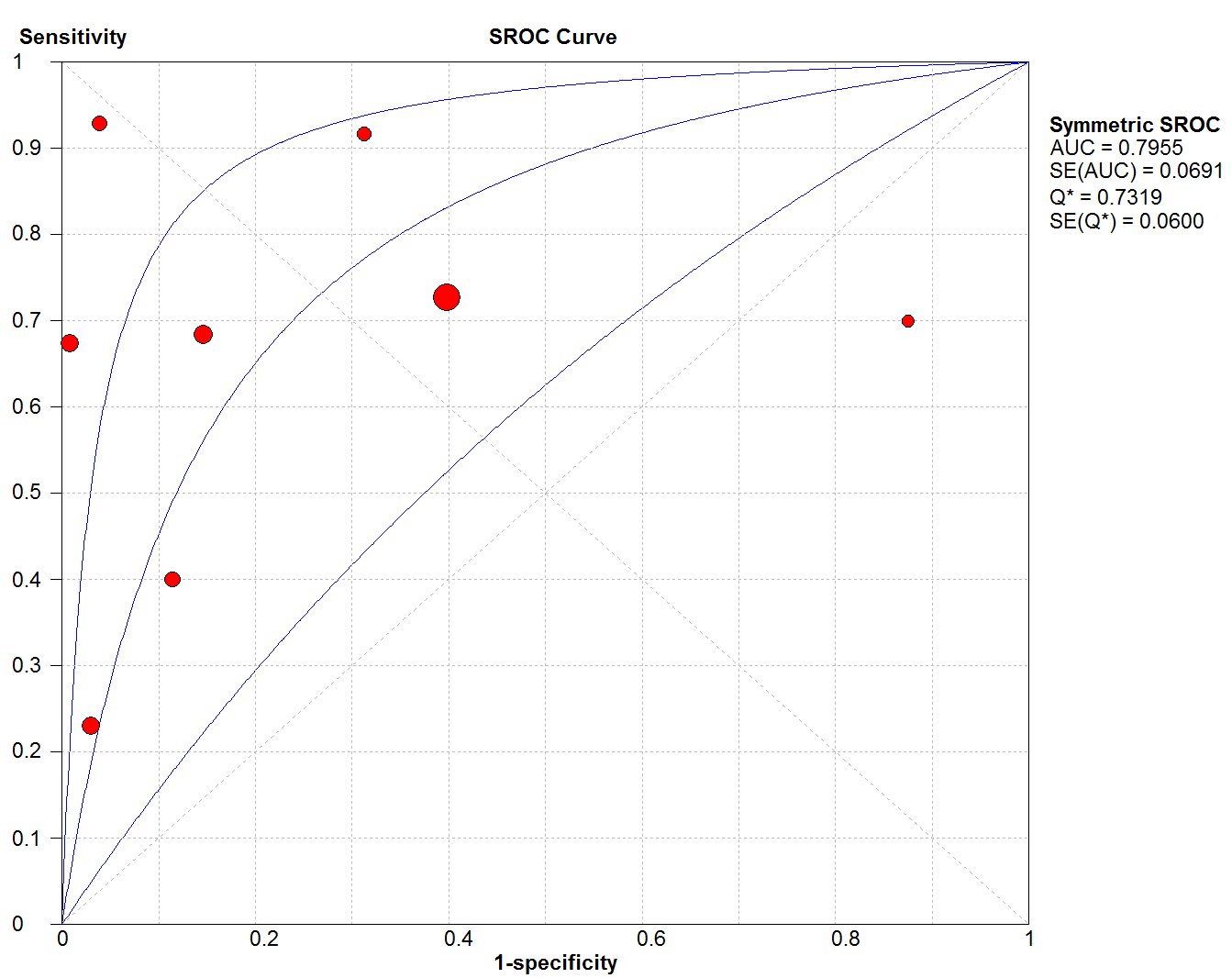
**

**E: CK-MB**

**
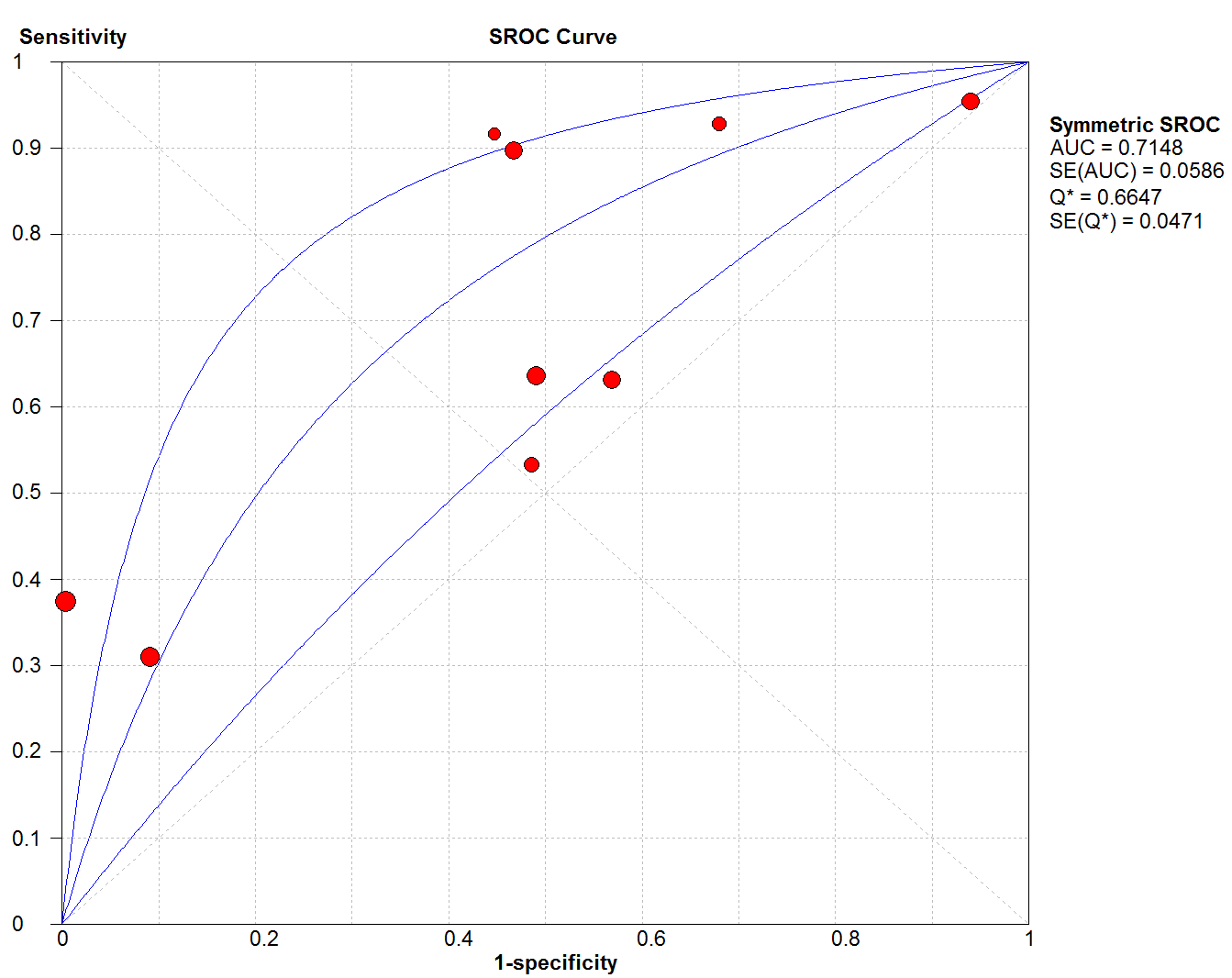
**
