# Supplementary material for: Diagnostic approach for myocardial contusion: a retrospective evaluation of patient data and review of the literature
Source: Eur J Trauma Emerg Surg. 2020 Jan 25;47(4):1259–72. doi: 10.1007/s00068-020-01305-4 (PMC8321993; doi:10.1007/s00068-020-01305-4)
Supplement: Supplementary file 2 — Supplementary file2 (DOCX 45 kb) [file 68_2020_1305_MOESM2_ESM.docx]

**Supplemental Table S1: Characteristics of all studies included in the literature review**

| **Manuscript** | **Study design** | **Population**  **(n)** | **Patients with myocardial contusion**  **(n)** | **Reference for myocardial contusion diagnosis** | **Mean age**  **(years)** | **IHD**  **(n)** | **AMI**  **(n)** |
| --- | --- | --- | --- | --- | --- | --- | --- |
| Burrell *et al.* (2017) (21) | Prospective | 42 | 21 | Troponin I elevation | 41 | N.S. | N.S. |
| Hammer *et al*. (2016) (41) | Retrospective | 42 | 42 | Clinical myocardial contusion | 52 | 12 | 0 |
| Mahmood *et al.* (2016) (42) | Prospective | 993 | 20 | Cardiac events | 30 | 0 | 0 |
| Sade *et al.* (2016) (14) | Prospective | 17 | 14 | ECG or echocardiography abnormality | 20-72* | 0 | 0 |
| Namrata *et al.* (2015) (43) | Prospective | 50 | 15 | Clinical myocardial contusion | N.S. | 0 | 0 |
| Bahar *et al.* (2014) (44) | Prospective | 210 | 47 | ECG or echocardiography abnormality | N.S. | 0 | 0 |
| Emet *et al.* (2010) (45) | Prospective | 88 | 22 | Cardiac events | 52 | 0 | 0 |
| Rajan *et al.* (2004) (35) | Prospective | 187 | 47 | Troponin I elevation and ECG abnormality | 38 | 0 | 0 |
| Velmahos *et al.* (2003) (23) | Prospective | 333 | 44 | Clinical myocardial contusion | N.S. | 0 | 0 |
| Mori *et al.* (2001) (46) | Prospective | 32 | 17 | Troponin I elevation | 44 | 0 | 0 |
| Salim *et al.* (2001) (47) | Prospective | 115 | 19 | Clinical myocardial contusion | N.S. | 0 | 0 |
| Bertinchant *et al.* (2000) (22) | Prospective | 94 | 26 | ECG or echo abnormalities | 51 | 0 | 0 |
| Boeken *et al.* (2000) (48) | Retrospective | 160 | 27 | ECG abnormality | 45 | 0 | 0 |
| Garcia-Fernandez *et al.* (1998) (49) | Prospective | 117 | 66 | Echocardiography abnormality | 37 | 0 | 0 |
| Ognibene *et al.* (1998) (50) | Prospective | 28 | 5 | Echocardiography abnormality | 44 | 0 | 0 |
| Swaanenburg *et al.* (1998) (51) | Prospective | 89 | 38 | Blunt thoracic trauma | 36 | 0 | 1 |
| Ferjani *et al.* (1997) (52) | Prospective | 128 | 29 | Clinical myocardial contusion | N.S. | 0 | 0 |
| Adams *et al.* (1996) (2) | Prospective | 44 | 6 | Wall motion abnormality | 54 | 0 | 0 |
| Batouk *et al.* (1996) (53) | Retrospective | 267 | 27 | Sternum fracture | 38 | 0 | 0 |
| Biffl *et al.* (1994) (36) | Prospective | 359 | 107 | Clinical myocardial contusion | 38 | 0 | 0 |
| Karalis *et al.* (1994) (54) | Prospective | 105 | 31 | Echo abnormality | 38 | 3 | 0 |
| Wisner *et al.* (1990) (8) | Prospective | 3,010 | 169 | Clinical myocardial contusion | N.S. | 0 | 0 |
| Baxter *et al.* (1989) (3) | Prospective | 280 | 35 | CK-MB elevation and ECG abnormality | N.S. | 0 | N.S. |
| Helling *et al.* (1989) (55) | Prospective | 68 | 49 | ECG or echocardiography abnormality or CK-MB elevation | 36 | 0 | 0 |
| Bodin *et al.* (1988) (56) | Prospective | 55 | 38 | Thallium-201 cardiac scanning | 35 | 0 | 0 |
| Fabian *et al.* (1988) (37) | Prospective | 140 | 56 | At risk after blunt thoracic trauma | 33 | 0 | 0 |
| Macdonald *et al*. (1981) (57) | Retrospective | 169 | 27 | ECG abnormality | 46 | 0 | 0 |
| Bayer *et al.* (1977) (58) | Prospective | 20 | 4 | Cardiac scanning | N.S. | 0 | 1 |

AMI, acute myocardial infarction; IHD, ischemic heart disease; N.S., not specified. * Range provided instead of mean.
